# Periprosthetic Femoral Fracture in Hip Arthroplasty Using Cemented Stems: Toward a Construct-Level and Time-Dependent Perspective

**DOI:** 10.3390/bioengineering13101222

**Published:** 2026-10-19

**Authors:** Mohamad Taufiqurrakhman, Kenzie Nathanael, Jules Deruelle, Hemant Pandit, Bernard van Duren

**Affiliations:** Leeds Institute of Rheumatic and Musculoskeletal Medicine (LIRMM), School of Medicine, University of Leeds, Leeds LS2 9JT, UK; kenzienathanael@gmail.com (K.N.); um23jpd@leeds.ac.uk (J.D.); h.pandit@leeds.ac.uk (H.P.); b.h.vanduren@gmail.com (B.v.D.)

**Keywords:** periprosthetic femoral fracture, cemented hip arthroplasty, polished taper-slip stem, cyclic loading, construct-level biomechanics

## Abstract

Periprosthetic femoral fracture (PFF) is an increasingly recognized complication following cemented total hip arthroplasty (THA); it is associated with substantial morbidity and mortality and often occurring several years after implantation. Although polished taper-slip (PTS) stems demonstrate favorable long-term survivorship, they have also been associated with a higher incidence of PFF, the biomechanical basis of which remains unclear. Existing studies have investigated a few key aspects of cemented THA, including implant migration, cement behavior, and fracture biomechanics. However, these factors are typically examined in isolation or under simplified conditions, and current construct-level models evaluate fracture without accounting for prior loading history. Pre-clinical testing standards relevant to fracture in cemented THA remain limited, with existing standards addressing individual components under simplified laboratory conditions rather than the integrated periprosthetic system under a physiologically relevant environment. As a result, how the implant–cement–bone construct evolves from implantation to eventual failure remains insufficiently characterized. In this study, we critically evaluate current experimental approaches and identify the absence of a time-resolved, construct-level framework as a key limitation. We outline a conceptual framework describing how implant migration, material response, and fracture behavior evolve with loading exposure. This framework may provide a foundation for both improving the mechanistic understanding of PFF and supporting the development of future pre-clinical testing strategies that can better reflect clinical loading conditions.

## 1. Introduction

Total hip arthroplasty (THA) is one of the most common surgical interventions, with over one million procedures performed globally each year [1]. However, periprosthetic femoral fracture (PFF) has emerged as a serious complication, occurring across the entire life cycle of THA after implantation and often following low-energy trauma such as a minor fall [2,3]. Its incidence is rising, and the reported one-year mortality exceeds 20%, similar to rates reported for native hip fractures in the elderly [4,5]. In the UK, approximately 1.6 million THAs were performed between 2003 and 2024, with about 116,900 procedures recorded in 2024 alone [6]. Around 60% of these involved full and hybrid cemented fixation, reflecting its continued widespread use, particularly in elderly and osteoporotic patients [3,7].

Cemented femoral fixation follows two primary design philosophies: composite beam (CB) stems which bond to the surrounding cement to function as a unified structure, and polished taper-slip (PTS) stems, which instead rely on controlled downward movement (subsidence) within the cement mantle to transfer load. PTS stems have demonstrated excellent long-term survivorship and low rates of aseptic loosening but are also associated with a higher incidence of PFF [7,8]. The biomechanical basis for this association remains unclear and is central to the question addressed in this manuscript.

Polymethylmethacrylate (PMMA) bone cement has been used in THA for over five decades and remains widely adopted due to its ability to conform to bone morphology and function as a load-transferring grout between the implant and bone [7,9]. Within the construct, it distributes load while accommodating micro-movements at the implant–bone interface during daily activity [7]. However, PMMA is a brittle polymer that accumulates fatigue damage under cyclic axial and torsional loading, with micro-crack initiation and propagation reported in both experimental and retrieved specimens [7]. This inherent property of PMMA should be considered, as such damage can progressively alter load transfer (hoop stresses and micromotion) within the cement mantle and its structural integrity over time [10,11,12]. With increasing life expectancy and activity levels in patient populations, cemented constructs may be subjected to cumulative mechanical loading, which may contribute to the rising incidence of PFF, among other factors [13,14].

The development of PFF may be governed by mechanical interactions between the implant, cement mantle, and surrounding bone under repeated loading, a coupled system hereafter referred to as the stem–cement–bone construct [15]. However, these interactions are difficult to isolate as current approaches do not adequately capture how construct behavior evolves prior to failure. This limitation extends to pre-clinical testing, where existing standards largely assess individual components, such as implant wear and fatigue or bone cement material properties, rather than the integrated stem–cement–bone construct under conditions relevant to fracture development [16].

**Figure 1:**
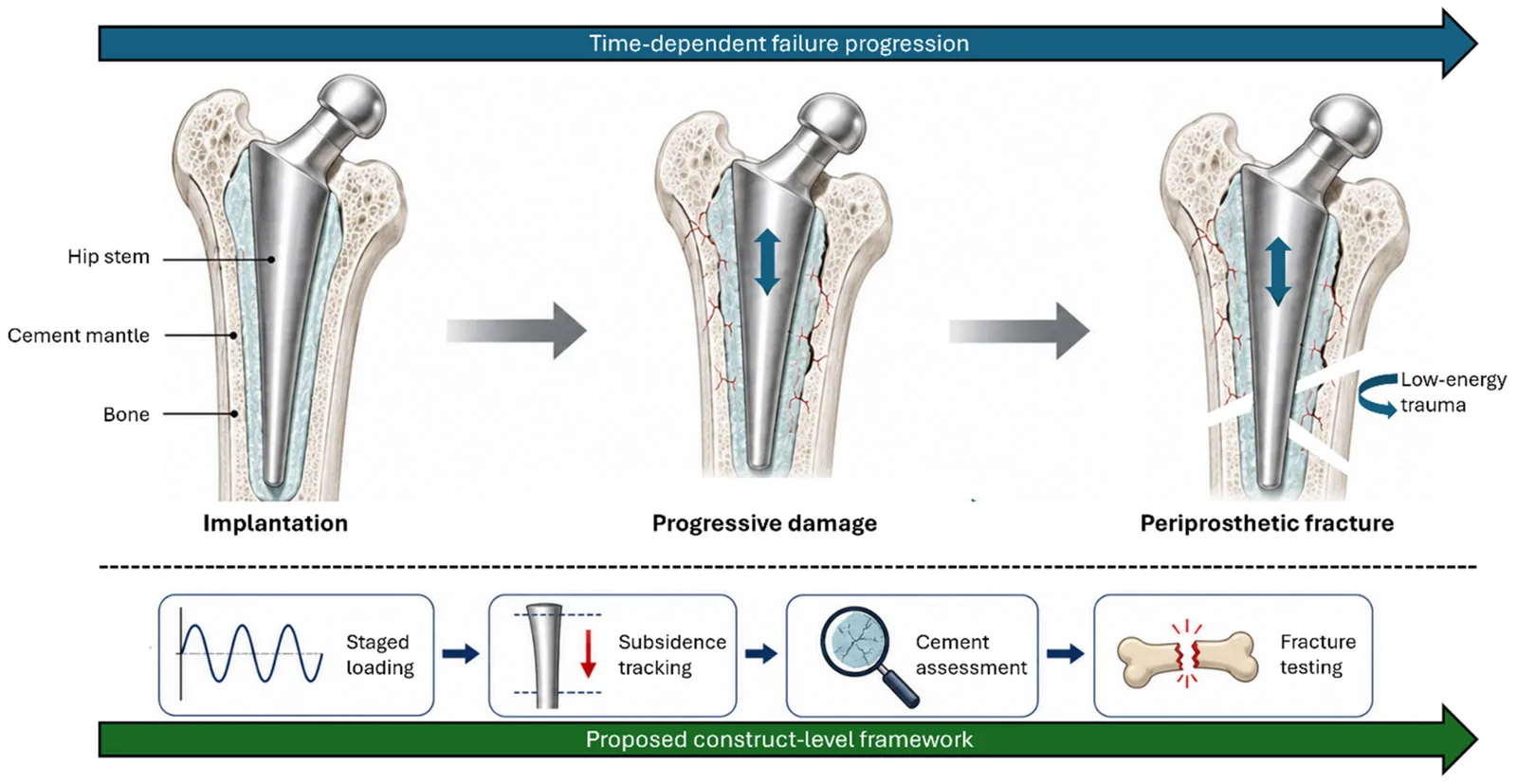
Conceptual overview of periprosthetic femoral fracture (PFF) as a time-dependent, construct-level failure process. Upper panel: The stem–cement–bone construct progresses from an intact post-implantation state, through cumulative damage accumulation in the cement mantle under cyclic loading, to eventual fracture triggered by low-energy trauma. Lower panel: The proposed experimental framework, consisting of staged cyclic loading, subsidence tracking, cement mantle assessment, and fracture testing, designed to resolve the relationships between mechanical exposure and construct-level outcomes.

In this manuscript, we argue that PFF in cemented THA should be understood not as an isolated fracture event but as the endpoint of a time-dependent failure process in the integrated stem–cement–bone construct, shaped by the progressive interaction of implant migration, cement behavior, loading history, and fracture response. Pre-clinical testing frameworks directly relevant to this problem remain limited with no standardized protocol in place, particularly at the implant–cement–bone construct level [17]. In this manuscript, we critically evaluate current experimental approaches, identify what is missing from existing pre-clinical testing, and propose a construct-level and time-dependent framework intended to assess not only implant performance, but also the integrity of the surrounding implant–cement–bone construct. This framework aims to improve our mechanical understanding of PFF development, while recognizing that fracture risk is also influenced by other factors, including age, bone quality, and mode of injury [3]. The conceptual basis of this perspective is summarized in Figure 1.

## 2. Current Understanding

This section outlines the current understanding of key components governing the biomechanical behavior of cemented THA. Rather than presenting the system in full complexity, it focuses on aspects that can be investigated at the construct level, including implant design and migration, cement mantle behavior, loading environment, and experimental testing approaches.

### 2.1. Stem Design and Periprosthetic Femoral Fractures

Cemented femoral stems are commonly classified into composite beam and polished taper-slip designs, representing two fundamentally different approaches to fixation and load transfer within the stem–cement–bone construct [7]. Early cemented stem designs largely followed the CB concept in which the stem bonds to the cement mantle so that the implant, cement, and surrounding bone function as a single structural unit [7,18]. Load is transferred through this composite system with minimal relative motion at the stem–cement interface [7].

Increasing clinical experience with these designs has revealed that CB stems are susceptible to aseptic loosening, which remains a leading cause of implant failure [7]. In response, an alternative design philosophy was introduced by Robin Ling and Clive Lee, characterized by a polished, collarless, tapered geometry designed to allow controlled subsidence within the cement mantle [7,19]. These PTS stems intentionally avoid bonding at the stem–cement interface, instead relying on relative downward motion to generate compressive radial forces that improve load transfer and reduce tensile stresses at the cement–bone interface [7].

This behavior is facilitated by a distal stem centralizer, which helps maintain a uniform cement mantle and allows the stem to subside in a controlled and centralized manner [18]. This migration pattern has been associated with favorable long-term fixation, with registry and clinical data consistently reporting lower revision rates for aseptic loosening in PTS compared to CB stems [7,18].

Although PTS stems have demonstrated improved resistance to aseptic loosening, this design shift has been associated with a different mode of failure: periprosthetic femoral fracture [7,18]. When compared to CB designs, PFF incidence in PTS stems was reported to be up to three times higher in low-risk patients and substantially higher in high-risk populations [8]. A recent meta-analysis reported incidence rate ratios ranging from 3.14 to 9.87 for postoperative PFF in PTS compared to CB stems, depending on the population studied [8]. The choice between CB and PTS stems can thus be viewed as a trade-off between different failure mechanisms, namely loosening versus fracture [7]. However, the higher reported PFF incidence in PTS stems may reflect not only a mechanical disadvantage but also longer implant survival with fewer early revisions for loosening, and differences in patient selection. Although the reported comparisons are based on incidence rates rather than simple proportions [8], registry and cohort date cannot account for the aforementioned residual confounders. Controlled laboratory comparison of the designs under identical conditions is one way to separate design effects from patient-related ones.

The biomechanical basis for the increased fracture risk associated with PTS stems is not yet fully understood. One hypothesis is that continued subsidence alters load transfer within the construct. As the tapered stem subsides axially, it generates radial compressive (hoop) stresses that are central to PTS fixation, as they improve load transfer and reduce tensile stress at the cement–bone interface [7]. It has not been determined whether, and under what conditions, these stresses become detrimental. A previous study suggested that this continued subsidence may raise hoop stresses in the cement mantle and surrounding cortical bone, enough to predispose the femur to longitudinal splitting under continued loading [12]. In addition, stress concentration from cracked bone cement may further increase local bone stress [10,20]. Combined with torsional loading during daily activity, this may contribute to the spiral fracture patterns commonly observed in PFF, although direct experimental evidence linking these mechanisms remains limited [21]. Conversely, controlled subsidence may be protective, and the overall effect may be related to the stage of loading.

In addition to design philosophy, parameters such as stem size, offset, and geometry may further influence load transfer and rotational behavior within the construct. Variations in these factors can modify stress distribution and have been suggested to affect fracture-related properties, particularly under torsional loading conditions [17,22,23,24,25,26]. Further research in this area alongside other factors that may contribute to PFF, such as age, bone quality, and mode of injury, may improve our understanding regarding the increased fracture risk associated with PTS stems [3].

### 2.2. Cement Mantle Behavior

PMMA bone cement functions as the primary load-transferring medium between the femoral stem and surrounding bone in cemented THA. Rather than forming a true adhesive bond, the cement acts as a grout, conforming to bone morphology and enabling load distribution across the stem–cement–bone construct [7].

The role of the cement mantle differs between CB and PTS designs. In CB stems, load is transferred through a bonded interface, with the cement acting as part of a unified structural system [7]. In contrast, controlled subsidence in PTS stems alter load transfer within the construct, with the cement accommodating both compressive loading and interfacial shear during implant migration [7,27].

At the material level, PMMA exhibits brittle behavior and is susceptible to fatigue under cyclic loading. Repeated mechanical loading has been shown to induce micro-crack initiation and propagation within the cement matrix, which may accumulate over time and reduce mechanical integrity [11]. In addition, intrinsic porosity within the cement, arising from mixing and curing processes, can also contribute to crack initiation under loading [10,11]. However, this has nuance, as some studies suggest that porosity alone may have limited effects on crack formation and require adjacent smaller voids or the presence of radio-pacifier particles to act as stress-risers [28]. A finite element model created by Janssen et al. also described the beneficial properties pores can provide in decelerating cracks and how heterogeneous in vivo stress fields may mask their influence, rendering them less intrinsically significant [29]. Although these models used modern vacuum mixing techniques, it is important to consider that mixing techniques have improved and reduced competing defects, so porosity may play a more prominent role. However, these ambivalent mechanistic properties still stand.

Recent µCT based studies have clarified the inverse relationship between porosity and bulk mechanical properties (i.e., compressive strength), but these tests remain constrained by ISO 5833 geometries and uniaxial loading [30]. As Barratt et al. emphasized, future work must evaluate PMMA performance in anatomically and mechanically realistic constructs, such as cemented femoral stems in composite femurs, to understand how porosity interacts with stem–cement and cement–bone interfaces under more clinically realistic loading conditions [30].

Within the construct, these material characteristics are influenced by factors such as cement mantle thickness, implant positioning, and loading conditions. The combined effect of cyclic loading, interfacial mechanics, and material heterogeneity leads to complex stress distributions within the cement mantle, particularly at the stem–cement and cement–bone interfaces, where damage initiation is most likely to occur [10,31]. Cement mantle geometry and quality are therefore not static background conditions but construct-level variables, with mantle thickness, centering, and porosity acting synergistically to influence both stress distribution and fatigue resistance under cyclic loading [10]. However, the relationship between mantle geometry and clinical performance is not straightforward. The so-called “French Paradox” refers to the observation that thin, line-to-line cementation techniques, which produce minimal cement mantles, demonstrate stable long-term clinical outcomes despite departing from the conventional recommendation of a 2 to 4 mm mantle [32]. This suggests that the mechanical interaction between stem, cement, and bone may matter more than mantle thickness alone, further supporting a construct-level rather than component-level interpretation of performance.

### 2.3. Loading Environment and Fracture Testing Approaches

In vivo, cemented hip constructs are subjected to complex and variable loading conditions during daily activities such as walking, stair climbing, and directional changes [33]. These activities generate combined axial and torsional loads, with peak joint contact forces reaching approximately two to three times that of body weight, accompanied by significant rotational moments at the femoral stem [33].

The combined loading conditions produce multiaxial stress states within the stem–cement–bone construct, influencing both implant migration and structural integrity [7]. PFFs associated with cemented stems commonly exhibit spiral or oblique fracture patterns, which are characteristic of torsional failure mechanisms [21]. Clinically, most of these fractures are classified as Vancouver type B2, occurring around the stem in the presence of a loose implant but with adequate surrounding bone stock [4,6,34]. The location and morphology of B2 fractures suggest that failure is governed by the interaction between axial loading, rotational forces, and construct-level instability rather than axial loading alone [21,34].

Construct-level biomechanical studies have been conducted to investigate these fracture patterns under controlled laboratory conditions aiming to recreate torsional fracture patterns as previously discussed. A commonly adopted experimental framework, first described by Morishima et al., involves applying combined axial compression and internal rotational loading to induce fracture [23]. This approach has been reproduced across multiple studies, including those by Ginsel et al., Takegami et al., and Jain et al., with variations in specimen type and loading configuration [17,22,24].

Reported outcome measures typically include torque to fracture, energy to fracture, rotation to failure, and fracture pattern, and more recent work has incorporated osteoporotic synthetic femora to better represent clinically relevant populations at higher risk of fracture [17,25]. Despite differences in methodology, these approaches have demonstrated consistent ability to reproduce clinically relevant fracture patterns, particularly Vancouver type B2 fractures [22,23,26].

## 3. Limitations of Current Approaches

Despite extensive investigation into cemented THA, the progression from initial loading to eventual fracture at the construct level remains incompletely characterized. Existing studies have examined key factors individually, including cement behavior, implant migration, and fracture mechanics, but have rarely evaluated these as coupled components of a single mechanical system. The following subsections examine three specific ways in which current approaches fall short, including limitations in testing standards, fragmentation across isolated mechanisms, endpoint-only construct models, and measurement resolution during testing.

### 3.1. Current Industry Standards

Mechanical characterization of PMMA bone cement is well established through standards such as ISO 5833 and ASTM F451/F2118 [35,36,37]. These tests provide reproducible measures of compressive strength, bending strength, and fatigue behavior using simplified specimen geometries and are used as a reference in many studies involving bone cement [11,16,30,38]. However, these conditions differ substantially from the in vivo environment, where the cement mantle is confined between the implant and bone while being subjected to multiaxial and interfacial loading [31]. Standardized cylindrical or rectangular specimens promote uniform stress distributions under uniaxial loading, which contrast sharply with the multiaxial, constrained stress states within the cement mantle. In vivo, confinement between stem and bone alters crack initiation and propagation pathways, producing complex gradients of compressive, tensile, and shear stresses [31]. An autopsy-based study by Race et al. described this well, but its main conclusions drew associations between crack propagation and implant duration or body mass index (BMI) rather than the stems themselves [39]. The interaction between the cement, implant, and surrounding bone plays a critical role in load transfer but is not yet represented in standard material tests. Although this is still useful or brand-to-brand comparison and describing mechanical properties, it makes the results of these standard tests less suitable for in-vivo applications.

In addition, standard cement testing is typically conducted under ambient laboratory conditions, whereas PMMA fatigue behavior is both temperature- and moisture-dependent. Testing in dry, room-temperature conditions can overestimate cement stiffness and underestimate damage accumulation relative to the physiological environment, further limiting the extent to which standard material properties can be extrapolated to in vivo construct behavior. Nottrott et al. demonstrated this directly, reporting no difference between specimens stored in water versus plasma, but significantly greater compressive strength in dry specimens compared to wet ones at all measured time points [11,36,38]. Ayre et al. further supported this point at the molecular level, where they described how the plasticizing effect of hydrolysis resulted in reduced mechanical and fatigue properties over time [11].

A similar limitation applies to implant-related testing. Existing standards for hip implants primarily address endurance of femoral components, wear of articulating bearing surfaces, or performance of modular interfaces, rather than the mechanical interaction between the femoral stem, cement mantle, and surrounding bone in cemented THA. For example, ISO 7206 evaluates the endurance properties of stemmed femoral components under specified laboratory conditions, ISO 14242 addresses wear testing of total hip joint prostheses at articulating surfaces, and ASTM F1875 focuses on fretting corrosion at the modular femoral head–taper interface [40,41,42]. As a result, current standards can assess individual components or selected interfaces in isolation but are not suitable for evaluating how stem–cement–bone interaction behaves and evolves under loading. Recent experimental frameworks have sought to bridge this gap through composite femur models and digital image correlation (DIC)-based strain mapping, enabling visualization of cement mantle deformation under physiological loading. For example, Mann et al. used DIC to show that creep deformation and crack initiation localize at the cement–bone interface [43,44,45]. Such approaches highlight that cement failure often initiates at interfacial stress concentrations rather than within the bulk material, underscoring the need for construct-level validation.

Collectively, these limitations indicate that standardized tests provide valuable baseline material properties but do not capture how the cement mantle behaves within the assembled construct under realistic loading conditions. A shift towards anatomically representative, multiaxial testing is required to understand how cement mantle integrity evolves under realistic loading and environmental conditions.

### 3.2. Construct-Level Models Capture Failure but Not Its Progression

Construct-level biomechanical models have been developed to investigate fracture behavior under controlled conditions, commonly using synthetic femoral models subjected to combined axial and rotational loading [17,22,23,24,25,26]. These models have demonstrated good repeatability and have successfully reproduced clinically relevant fracture patterns, particularly Vancouver type B2 fractures [22,23,26].

However, these experiments typically evaluate fracture under monotonic loading without prior cyclic exposure. For example, Takegami et al. conducted such construct-level testing to demonstrate that PTS stems were more susceptible to PFF, reinforcing a widely cited limitation of the PTS design relative to CB stems [24]. However, this interpretation may be constrained, as fracture occurs after prolonged periods of functional loading, during which implant migration, interface changes, potential material degradation, or even improvements in construct integrity due to stabilizing hoop stresses from the PTS design may develop [2,3,24,46]. Current models assess fracture at a single endpoint rather than after defined stages of mechanical exposure and therefore cannot capture how the construct transitions from initial implantation to eventual failure. Clinically, PFF typically occurs several years after implantation, making the absence of prior cyclic exposure in current models a particularly significant limitation [2,3].

Direct evidence that prior cyclic loading alters construct-level fracture resistance is currently lacking, which is the research gap addressed in this manuscript. Indirect evidence suggests it may: PMMA accumulates fatigue damage and loses mechanical properties with moisture uptake and aging [11,39], damage accumulation correlated with duration of use has been observed in cement mantles retrieved at autopsy [38], and fatigue-creep damage develops at the cement–bone interface [10,45]. However, these findings arise from material interface or retrieval studies rather than fracture testing of loaded constructs, and their net effect on fracture resistance could be adverse, neutral, or protective. Identifying the direction and magnitude of this effect is a central aim of this proposed framework.

This limitation is partly methodological. Implant migration, particularly subsidence, is a defining characteristic of PTS stems and is commonly assessed using radiographic or radio stereometric methods in clinical settings [46]. These techniques provide high accuracy but are not readily integrated into mechanical testing and typically yield discrete measurements rather than continuous data. In laboratory settings, displacement is sometimes inferred from the outputs of the testing apparatus, which represent global system movement and may include contributions from compliance and fixture deformation [47]. This limits the ability to quantify local implant movement within the construct during loading. Contemporary radio-stereometric analysis (RSA) studies of cemented taper slip stems, such as that by Nieuwenhuijse et al., have characterized long-term migration patterns of the Exeter stem, demonstrating continued subsidence and retroversion over the first decade after implantation [48]. However, while RSA provides detailed information on stem movement, it does not evaluate how this migration relates to concurrent changes in cement mantle integrity or evolving fracture susceptibility within the same construct. Moreover, cement mantle fractures were only identified when visible on radiographs, and RSA could not determine whether migration occurred at the prosthesis–cement or cement–bone interface. As a result, migration behavior is quantified independently of the material and interfacial responses that ultimately govern construct-level failure.

Alternative approaches, including the use of micro-computed x-ray tomography (micro-CT) and linear variable differential transformers (LVDTs), better represent movements within the bone–implant construct, but each carries its own constraints [19,49]. Micro-CT is often time- and resource-intensive, while LVDTs may compromise structural integrity and require physical access to the construct, potentially affecting subsequent biomechanical testing.

Both the conceptual design of current models and the practical difficulty of resolving migration during loading mean that it is currently not possible to track how the construct evolves from implantation toward fracture using construct-level testing.

### 3.3. Key Mechanisms Are Evaluated in Isolation

The key factors associated with cemented THA performance, including implant subsidence, cement fatigue, and fracture mechanics, have been widely studied. However, no existing studies have tracked migration, cement integrity, and fracture resistance across defined loading histories within a single experimental series. These factors have typically been investigated independently using different experimental or analytical approaches rather than as interacting components of a single stem–cement–bone construct [7,11,17,22,23,24,25,26]. As a result, current approaches do not allow for drawing a direct correlation between migration, material response, and structural failure within a single experimental system. It therefore remains unclear how these mechanisms interact over time or whether they contribute sequentially to fracture development. Many representative studies indicate this. Construct-level fracture models evaluate fracture response in synthetic femora without prior cyclic loading or concurrent measurement of migration or cement damage [17,21,22,23,24,25]. A clinical RSA study characterized migration over a decade but only detected cement cracks when visible on radiographs; it does not relate migration to fracture susceptibility [48]. Autopsy retrievals link cement damage to duration of use but do not measure migration or fracture resistance. Interface-level studies characterize fatigue and creep damage at the cement–bone interface in simplified specimens rather than assembled constructs [10,30,45]. Each study evidently captures one or two of the relevant variables, but none combines them with a defined loading history. However, this perspective is based on a narrative, non-systematic review of the literature.

## 4. Toward a Construct-Level Framework

The limitations identified in the previous section highlight a consistent gap: the absence of an experimental framework that resolves how the stem–cement–bone construct evolves under loading rather than how it behaves at a single point in time. Addressing this requires a framework in which the same construct is tracked progressively, and in which migration, material response, and fracture behavior are treated as coupled outcomes rather than independently studied phenomena.

The framework is motivated by the PTS-PFF association but is not exclusive to PTS stems. Its components can be applied to CB stems, where relevant migration modes and damage mechanisms differ sustained subsidence versus debonding at the stem–cement interface, respectively. Applying an identical protocol to both designs may be the most direct test of whether PTS-associated fracture risk reflects design-specific behavior. Cement-specific measures would not transfer to cementless fixation, although staged loading and fracture endpoints could.

In this manuscript, we propose a time-resolved, construct-level framework built around four categories of measurable parameters: implant migration, construct-level mechanical response, cement mantle integrity, and fracture response. Implant migration should be tracked continuously or at defined cyclic intervals, with particular attention paid to subsidence and rotational movement in polished taper-slip stems, as these are the migrations most directly relevant to load-transfer behavior [33]. In practice, this can be achieved using LVDTs or marker-based visual tracking methods, both of which are available in standard biomechanics laboratories. LVDTs provide direct, high-resolution measurements of implant displacement and are well suited for quantifying axial subsidence during cyclic loading, but their physical attachment may require setup modifications to prevent interference with subsequent fracture testing [50]. In contrast, marker-based visual tracking is non-contact and less mechanically intrusive, although its accuracy depends on adequate camera resolution, calibration, and marker visibility [51]. Construct-level mechanical responses including stiffness and hysteresis behavior (energy dissipated per loading cycle) should be measured periodically throughout loading to identify whether the construct stabilizes or transitions as loading accumulates. These can be derived directly from the load–displacement data recorded by the testing apparatus, requiring no additional equipment. Cement mantle integrity should be assessed both during and after loading, ideally through micro-CT imaging at staged intervals and post-test sectioning to characterize micro-crack initiation, propagation, and interface condition at both the stem–cement and cement–bone interfaces [49]. Micro-CT enables non-destructive 3D assessment of cement damage and interfacial defects while preserving the construct for subsequent fracture testing, but its detection of fine microcracks may be limited by voxel resolution and metal artifacts. Meanwhile, post-test sectioning, especially when combined with techniques such as optical microscopy or scanning electron microscopy (SEM), can provide higher-resolution visualization of fine cracks and interface morphology but is destructive and should only be used for endpoint characterization. The use of micro-CT also requires access to dedicated imaging facilities but is widely available in university and hospital research settings. Where available, digital image correlation (DIC) may provide additional surface strain data during loading, though this is not essential to the framework. Fracture response, quantified through torque to fracture, energy to fracture, rotation at failure, and fracture pattern, should be measured after defined loading histories rather than from the native post-implantation state so that fracture behavior can be interpreted in relation to earlier loading and aging [17,22,23,24,25]. This can be performed using standard servo hydraulic testing systems following established protocols.

**Figure 2:**
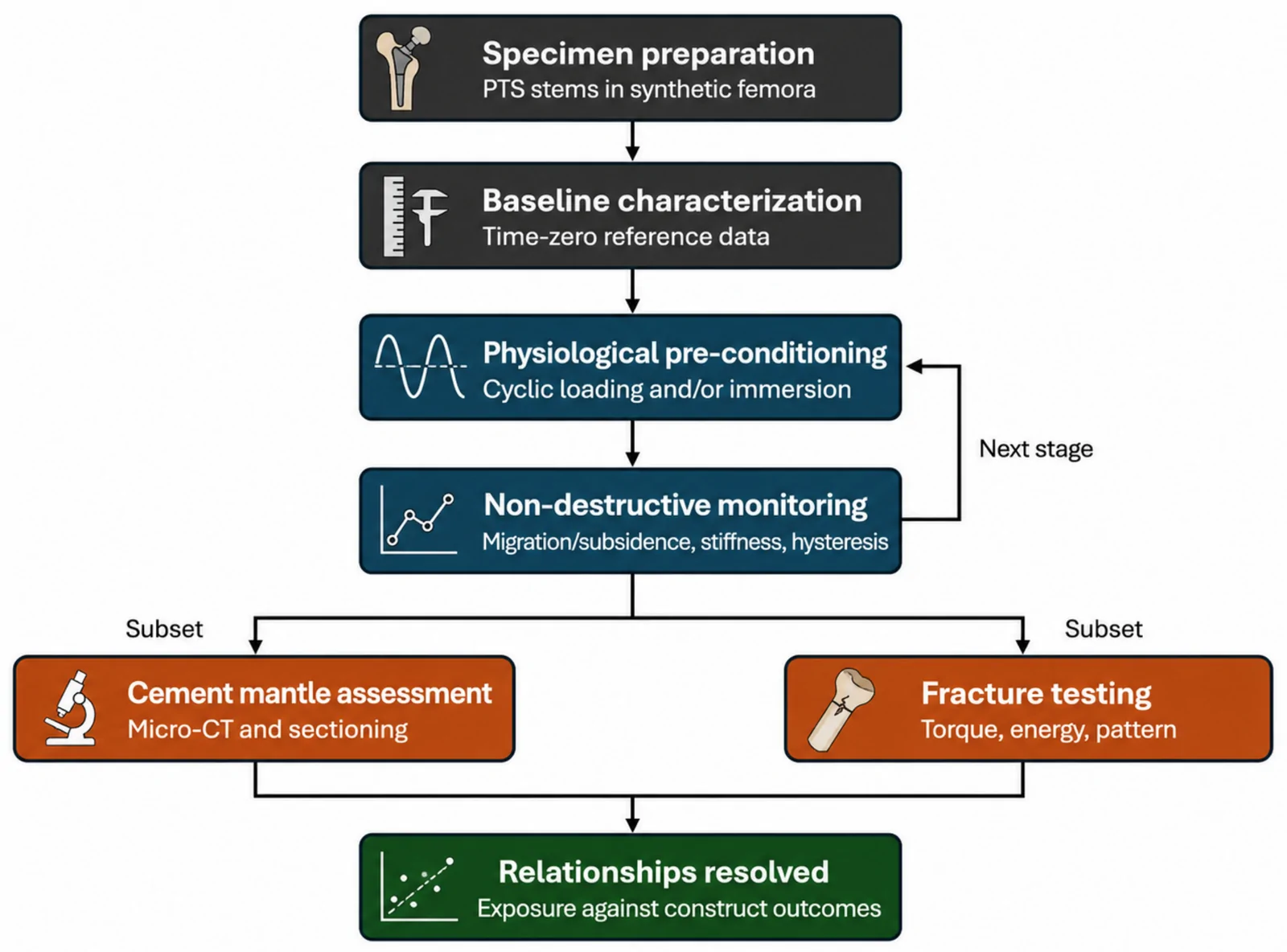
Proposed staged experimental workflow for construct-level investigation of periprosthetic femoral fracture. Cemented polished taper-slip femoral stems are implanted in synthetic femora and characterized at baseline (time zero). Constructs undergo physiological pre-conditioning through cyclic loading and/or environmental immersion in discrete staged blocks, with non-destructive monitoring of migration, stiffness, and hysteresis between each block. At defined stages, subsets of constructs are removed for cement mantle assessment (micro-CT and sectioning) or fracture testing (torque to fracture, energy to fracture, and fracture pattern). The loop between pre-conditioning and monitoring enables fracture resistance and material degradation to be evaluated as functions of cumulative exposure.

A realistic in vitro implementation example of this framework is outlined in Figure 2. Cemented polished taper-slip femoral stems would be implanted in synthetic femora (normal or osteoporotic) or cadaveric femora using standardized cementing technique, with mantle thickness, centering, and porosity controlled as construct-level variables. Prior to any loading or environmental exposure, all constructs would undergo baseline characterization at time zero, including initial construct stiffness, migration reference datum (axial and rotational), and baseline micro-CT of the cement mantle, to document initial porosity distribution and interface condition. Synthetic femora provide consistent bone quality and withstand prolonged testing, making them suitable for mapping and comparing loading trajectories. Their limitations are the absence of biological tissue behavior and anatomical variation. Furthermore, a cancellous analog cannot perfectly reproduce cement interdigitation with human trabecular bone, which is relevant to cement–bone interface damage. Cadaveric femora better reproduce anatomical variation and the interface but are more variable, costly, and difficult to preserve. We therefore propose initial testing in synthetic femora followed by cadaveric validation at selected stages. Bone quality will be treated as a fixed between-specimen factor, allowing for assessment of its interaction with loading exposures.

Synthetic models should not be assumed to reproduce human bone behavior. Their validity can be assessed by (i) cadaveric validation at selected stages and conditions, (ii) comparison of fracture patterns and torques with cadaveric and clinical data [22,23,24,26,27], (iii) comparison of subsidence magnitude and rate with clinical RSAs [46,48], and (iv) comparison of cement damage patterns with retrieval studies [38,39]. Direct validation of accelerated loading against in-vivo behavior is not possible, so these benchmarks only provide face validity.

Constructs would then be subjected to single or combined physiological pre-conditioning protocols applied in discrete staged blocks. Mechanical pre-conditioning consists of combined cyclic axial and torsional loading at physiological magnitudes, reflecting activities such as walking, stair climbing, and pivoting [21,33]. Environmental pre-conditioning consists of immersion in phosphate-buffered saline at 37 °C for defined durations to simulate moisture uptake and cement aging [16,38,39]. These can be applied independently or in combination to create testable subgroups (loading only, immersion only, both, and untreated baseline). The loading protocol itself is a framework variable, as different activity profiles generate different axial–torsional combinations and different damage trajectories.

Stages should be defined by approximate clinically relevant loading exposure. Patients with well-functioning THAs have been reported to undertake a mean of 2.24 million walking cycles annually, although activity varies substantially with patient characteristics [52]. On this basis, each block of 106 cycles represents approximately 5 to 6 months of average walking exposure. Four to five assessment points, comprising baseline and blocks at 1, 2, 5, and 10 years, for example, would span the first decade, over which taper-slip stems continue to subside [46,48]. Denser early sampling would be performed where migration is fastest [46]. Cycle frequency should be low enough to avoid heating the cement. The cycle-to-year equivalence is approximate, and creep and moisture uptake do not scale with cycle count, which is why environmental conditioning is applied as a separate, time-based factor.

Between each loading block, all constructs undergo non-destructive measurement windows during which implant migration (axial subsidence and rotation), construct stiffness, and hysteresis behavior are recorded [19,46,47,49,53]. These parameters are tracked cumulatively across stages to capture the progressive evolution of construct response. At defined stages, a subset of constructs is removed from the loading sequence for detailed cement mantle evaluation, including micro-CT imaging to map micro-crack distribution and porosity changes, and sectioning to characterize crack propagation and interface condition at both the stem–cement and cement–bone interfaces [49]. These results are compared against the time zero baseline to quantify loading-induced damage. At the same stages, a separate subset of constructs undergoes fracture testing under combined axial compression and torsional loading, with outcome measures including torque to fracture, energy to fracture, rotation at failure, and fracture pattern [17,22,23,24,25,26,27]. Because fracture testing is performed at multiple stages rather than only at time zero, fracture resistance can be interpreted as a function of prior mechanical and environmental exposure. Although more demanding than single-endpoint testing, this kind of staged design is feasible and offers a practical starting point for future construct-level studies.

The framework can be implemented incrementally with a minimal set-up requiring an axial–torsional test frame (providing stiffness and hysteresis from load–displacement data), a local displacement measurement (LVDT or marker-based tracking), and staged fracture testing. Micro-CT, DIC, and environmental conditioning are complementary additions where facilities permit. Because destructive endpoints cannot be repeated on a single construct, the sample size will be scaled in line with the number of stages. For example, a baseline plus three loading stages, with six constructs per fracture group and three per micro-CT/sectioning group, would require approximately 33 constructs per test condition; formal sample size calculations should be conducted based on variances identified in pilot studies. This is evidently more demanding than single-endpoint testing, but the use of synthetic femora (low inter-specimen variability and the restriction of micro-CT to staged subsets) keeps it feasible for many well-equipped laboratories.

The framework could be used to explore relationships that current isolated or single-endpoint approaches cannot resolve: (a) subsidence magnitude and rate versus cycle number, (b) construct stiffness evolution versus loading stage, (c) torque to fracture and energy to fracture versus prior mechanical and environmental exposure, (d) cement mantle damage versus loading stage, and (e) fracture resistance versus cement integrity at each corresponding stage. Together, these relationships may provide a better mechanistic basis for identifying which aspects of construct degradation govern the transition toward PFF. Laboratory timescales cannot fully replicate the duration and continuity of in vivo loading, and any such framework is necessarily an accelerated approximation. Even so, establishing these relationships would reframe fracture as a progressive process rather than a single endpoint. Examples of questions that the framework is designed to address include: (i) whether any loss of fracture resistance is associated with cumulative subsidence, cement damage, or neither; (ii) whether fracture resistance steadily changes in one direction with regard to loading exposure, or whether it increases (stabilizing hoop stress mechanism) before declining; (iii) whether cement damage precedes, follows, or is independent of changes in construct stiffness and hysteresis; and (iv) whether PTS and CB designs follow different trajectories under identical loading exposure conditions. Because these questions concern sequence and interactions, they cannot be resolved by pooling results from separate studies that differ in specimens, cement, loading, and boundary conditions.

## 5. Implications and Future Directions

Reframing PFF as a time-dependent, construct-level failure process has implications beyond biomechanical interpretation. It may shift the basis on which cemented hip constructs are evaluated, from single-point fracture testing toward assessment of how construct behavior develops under realistic loading exposure. This opens the possibility of interpreting pre-clinical results mechanistically, linking observed failure to the prior evolution of migration and material response rather than to the fracture event in isolation.

For pre-clinical testing, this perspective suggests a direction in which future standards might complement existing component-level tests with construct-level evaluation that incorporates loading history. Such an addition would not replace current material or implant standards but would address the system-level behavior that those standards are not designed to capture. This may support a more informed comparison between cemented stem designs, cement formulations, and construct configurations in relation to their behavior under clinically relevant loading.

The proposed framework addresses the mechanical contribution to PFF and should not be read as a model of the full clinical process. Accelerated laboratory loading cannot reproduce the biological and temporal conditions under which PFF develops in vivo, including bone remodeling, age-related and peri-implant bone loss, osteolysis, and the variability of patient comorbidities and activities. In addition, cycle count does not scale all time-dependent processes such as cement creep and moisture uptake, which is why environmental conditioning is treated as a separate factor. Findings are therefore most appropriately interpreted comparatively (e.g., between stem designs, cement conditions, or bone quality) and as hypothesis-generating rather than as predictions of absolute fracture risk or time to fracture. Absolute conclusions should be triangulated alongside in vivo studies including RSA, retrieval analyses and registry data.

Future experimental work may benefit from developing and validating approaches consistent with this proposed framework, including reproducible staged loading protocols, continuous or near-continuous migration measurement, and methods for quantifying cement mantle damage within intact constructs. The framework proposed in this manuscript addresses the mechanical dimension of PFF development whereas in vivo fracture also involves biological processes such as bone remodeling and peri-implant bone loss, which future integrated studies may need to consider alongside construct-level mechanics. Progress in these areas would provide the foundation for a more holistic understanding of PFF, and ultimately for pre-clinical testing strategies that better reflect the conditions under which these fractures develop in patients.

## Data Availability

No new data were created or analyzed in this study. Data sharing is not applicable to this article.

## Abbreviations

The following abbreviations are used in this manuscript:

PFF	Periprosthetic femoral fracture
THA	Total hip arthroplasty
CB	Composite beam
PTS	Polished taper-slip
PMMA	Polymethylmethacrylate
BMI	Body mass index
ISO	International Organization for Standardization
ASTM	American Society for Testing and Materials
DIC	Digital image correlation
RSA	Radio-stereometric analysis
microCT	Micro-computed tomography
LVDT	Linear variable differential transformer
SEM	Scanning electron microscopy
